# Phenazin-5-ium hydrogen sulfate monohydrate

**DOI:** 10.1107/S160053681300562X

**Published:** 2013-03-02

**Authors:** Joseph deGeorge, Christopher P. Landee, Mark M. Turnbull

**Affiliations:** a Department of Physics, Clark University, 950 Main St, Worcester, MA 01610, USA; b Department of Chemistry, Clark University, 950 Main St, Worcester, MA 01610, USA

## Abstract

The crystal structure of the title salt, C_12_H_9_N_2_^+^·HSO_4_^−^·H_2_O, comprises inversion-related pairs of phenazinium ions linked by C—H⋯N hydrogen bonds. The phenazinium N—H atoms are hydrogen bonded to the bis­ulfate anions. The bis­ulfate anions and water mol­ecules are linked by O—H⋯O hydrogen-bonding inter­actions into a structural ladder motif parallel to the *a* axis.

## Related literature

For related structures, see: Sieroń (2007 ▶) [phenazinium perchlorate]; Plasseraud *et al.* (2009 ▶) [phenazinium trifluoro­methane­sulfonate]; Braga *et al.* (2010 ▶) [phenazinium chloride and phenazine monohydrate]; G.-X. Zhang *et al.* (2012 ▶) [phenazinium bromide]; N.-Q. Zhang *et al.* (2012 ▶) [phenazinium methane­sulfonate]. For copper(II) salts of phenazine, see: Schneider *et al.* (2007 ▶). For graph-set analysis of hydrogen bonding, see: Bernstein *et al.* (1995 ▶).
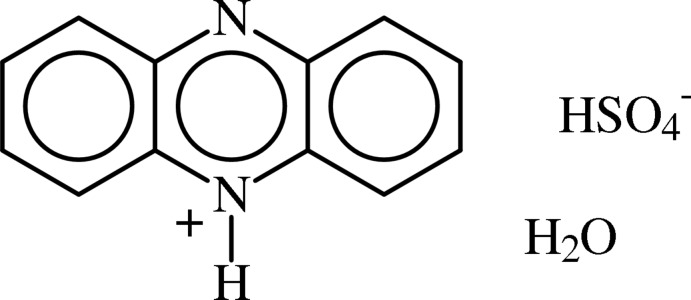


## Experimental

### 

#### Crystal data

C_12_H_9_N_2_^+^·HSO_4_^−^·H_2_O
*M* *_r_* = 296.30
Triclinic, 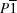

*a* = 5.6565 (4) Å
*b* = 10.4019 (6) Å
*c* = 10.9500 (5) Å
α = 89.693 (4)°
β = 87.202 (5)°
γ = 76.412 (5)°
*V* = 625.49 (6) Å^3^
*Z* = 2
Cu *K*α radiation
μ = 2.53 mm^−1^
*T* = 120 K
0.45 × 0.40 × 0.30 mm

#### Data collection

Agilent SuperNova (Dual, Cu at zero, Atlas) diffractometer
Absorption correction: multi-scan (*CrysAlis PRO*; Agilent, 2011 ▶) *T*_min_ = 0.786, *T*_max_ = 1.000
3789 measured reflections
2341 independent reflections
2276 reflections with *I* > 2σ(*I*)
*R*_int_ = 0.013

#### Refinement

*R*[*F* ^2^ > 2σ(*F* ^2^)] = 0.032
*wR*(*F* ^2^) = 0.088
*S* = 1.09
2341 reflections
193 parameters
H atoms treated by a mixture of independent and constrained refinement
Δρ_max_ = 0.30 e Å^−3^
Δρ_min_ = −0.39 e Å^−3^

### 

Data collection: *CrysAlis PRO* (Agilent, 2011 ▶); cell refinement: *CrysAlis PRO*; data reduction: *CrysAlis PRO*; program(s) used to solve structure: *SHELXTL* (Sheldrick, 2008 ▶); program(s) used to refine structure: *SHELXTL*; molecular graphics: *SHELXTL* and *Mercury* (Macrae *et al.*, 2008 ▶); software used to prepare material for publication: *SHELXL97* (Sheldrick, 2008 ▶), *enCIFer* (Allen *et al.*, 2004 ▶) and *publCIF* (Westrip, 2010 ▶).

## Supplementary Material

Crystal structure: contains datablock(s) I, global. DOI: 10.1107/S160053681300562X/sj5299sup1.cif

Structure factors: contains datablock(s) I. DOI: 10.1107/S160053681300562X/sj5299Isup2.hkl

Supplementary material file. DOI: 10.1107/S160053681300562X/sj5299Isup3.cml

Additional supplementary materials:  crystallographic information; 3D view; checkCIF report

## Figures and Tables

**Table 1:** Hydrogen-bond geometry (Å, °)

*D*—H⋯*A*	*D*—H	H⋯*A*	*D*⋯*A*	*D*—H⋯*A*
N1—H1⋯O1	0.86 (2)	1.81 (2)	2.6685 (18)	173.3 (19)
O2—H2⋯O1*S*	0.93 (2)	1.59 (2)	2.5223 (16)	177 (2)
O1*S*—H1*A*⋯O4^i^	0.89 (2)	1.87 (2)	2.7577 (18)	176 (2)
O1*S*—H1*B*⋯O3^ii^	0.84 (2)	1.90 (2)	2.7405 (18)	173 (2)
C6—H6⋯N8^iii^	0.93	2.61	3.538 (2)	172

Symmetry codes: (i) 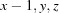; (ii) 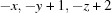; (iii) 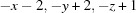.

**Fig. 1.**
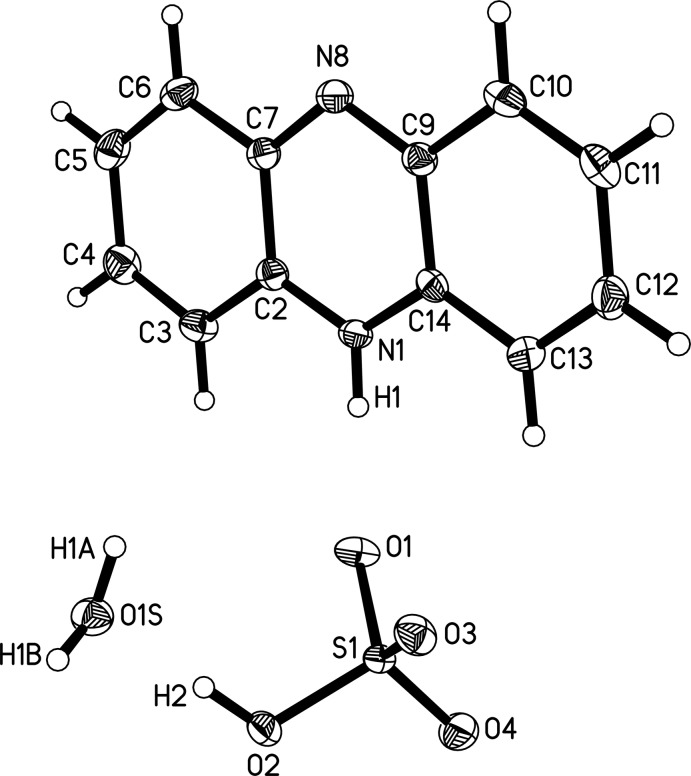
Thermal ellipsoid plot (50% probability) of compound 1. Hydrogen atoms are shown as spheres of arbitrary size and only hydrogen atoms whose positions were refined are labeled.

**Fig. 2.**
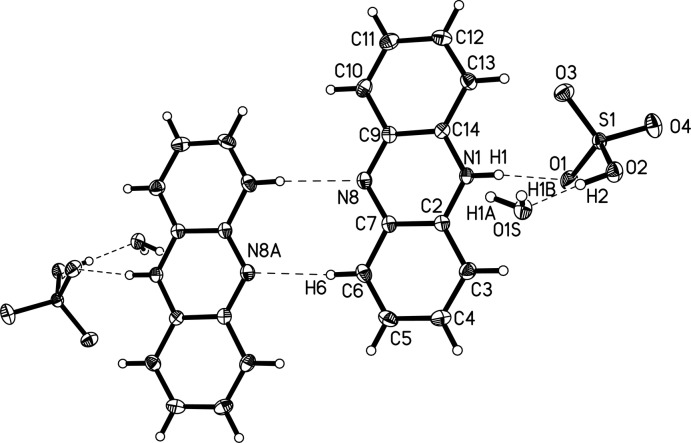
Thermal ellipsoid plot of 1 (50% probability) showing two inversion related asymmetric units. Only those H-atoms involved in hydrogen bonding are labeled. Hydrogen atoms are shown as spheres of arbitrary size.

**Fig. 3.**
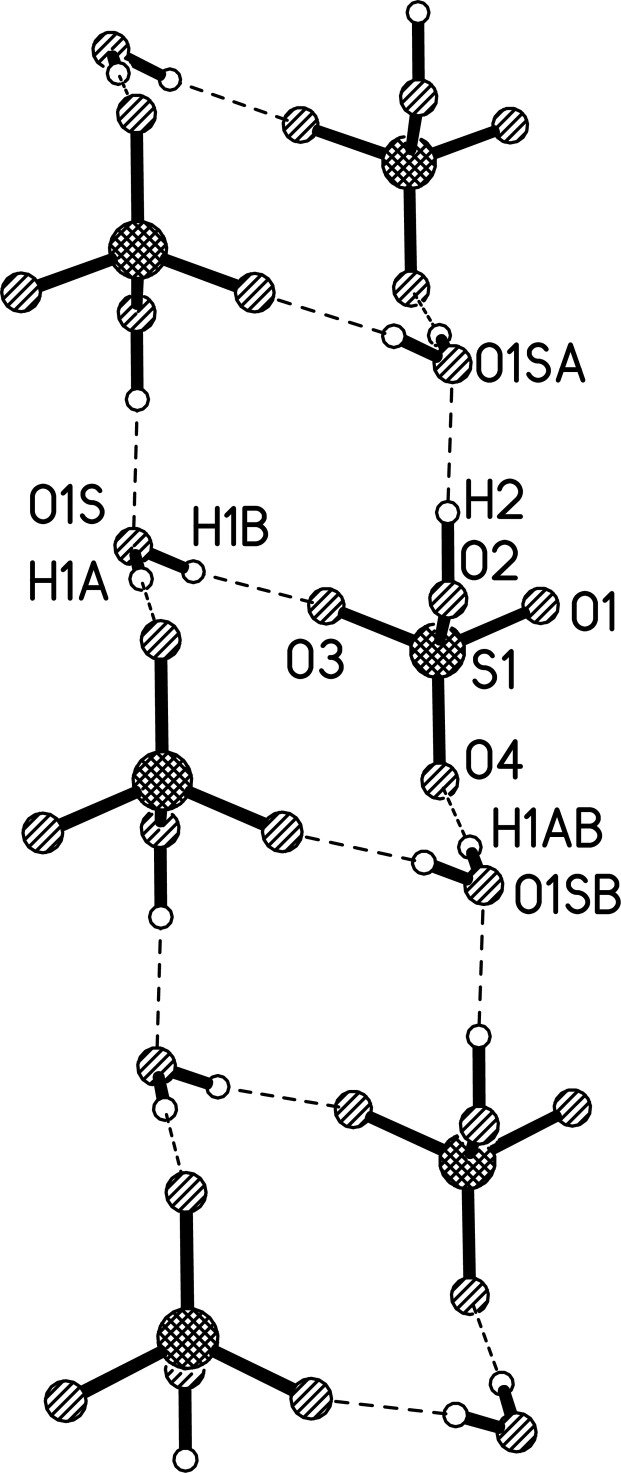
Packing diagram showing the structure of the ladder motif formed by hydrogen bonding between the bisulfate ions and water molecules. Details of the hydrogen bonding may be found in Table 1.

## supplementary crystallographic information

### Comment

Phenazinium bisulfate monohydrate (Figure 1) crystallized originally as a biproduct of our work with copper (II) salts of phenazine (Schneider, *et al.*, 2007). The phenazinium ions crystallize as weakly hydrogen bonded centrosymmetric dimers (see Figure 2), forming *R*^2^_2_(8) rings (Bernstein *et al.*, 1995), similar to the structures of the chloride [Braga, *et al.*, 2010] and perchlorate salts [Sierón, 2007] and to the phenazinium trifluoromethanesulfonate:phenazine co-crystal [Plasseraud, *et al.*, 2009]. The phenazinium proton is involved in a strong hydrogen bond to one of the bisulfate oxygen atoms [d_D···A_ = 2.6685 (18) Å] (see Figure 1). The phenazinium rings are stacked parallel to the *a*-axis with a distance of 3.9156 (9) Å between the ring centroids of the diazine rings and a slip angle of 30.1°.

Perhaps the most interesting aspect of the structure results from the hydrogen bonding between the bisulfate anions and the solvent water molecule. This results in the formation of a ladder motif that runs parallel to the *a*-axis (see Figure 3). Each bisulfate ion serves as a hydrogen bond donor to one water molecule and a hydrogen bond acceptor from a second water molecule forming the rails of the ladder, of form C^2^_2_(6). The rungs are formed *via* a second water-donor/bisulfate-acceptor pair, which generates rings within the ladder structure (two rungs and two rail sections in each ring), *R*^4^_4_(12). There are two chemically different rings formed in this case since one involves rail sections with water molecules serving as the hydrogen bond donor and the other involves the bisulfate ion serving as the hydrogen bond donor.

### Experimental

Phenazine was dissolved methanol (90 ml) to which 40% aqueous sulfuric acid (2.5 ml) had been added. Small, prismatic, ruby red crystals formed over the course of two months of slow evaporation at room temperature.

### Refinement

All H-atoms bound to carbon were refined using a riding model with d(C—H) = 0.93 Å, *U*_iso_=1.2*U*_eq_ (C). Hydrogen atoms bonded to oxygen or nitrogen atoms were located in a difference map and their positions refined using fixed isotropic U values. There are two Level-B warnings in the checkCIF file for short intermolecular H···H distances. These result from the very strong hydrogen bond between the bisulfate ion and the solvent water molecule (d_D···A_ = 2.5223 (16) Å.

### Crystal data

**Table:**

C_12_H_9_N_2_^+^·HSO_4_^−^·H_2_O	*Z* = 2
*M**_r_* = 296.30	*F*(000) = 308
Triclinic, *P*1	*D*_x_ = 1.573 Mg m^−^^3^
Hall symbol: -P 1	Cu *K*α radiation, λ = 1.54184 Å
*a* = 5.6565 (4) Å	Cell parameters from 3015 reflections
*b* = 10.4019 (6) Å	θ = 4.0–73.7°
*c* = 10.9500 (5) Å	µ = 2.53 mm^−^^1^
α = 89.693 (4)°	*T* = 120 K
β = 87.202 (5)°	Prism, red
γ = 76.412 (5)°	0.45 × 0.40 × 0.30 mm
*V* = 625.49 (6) Å^3^	

### Data collection

**Table:**

Agilent SuperNova (Dual, Cu at zero, Atlas) diffractometer	2341 independent reflections
Radiation source: SuperNova (Cu) X-ray Source	2276 reflections with *I* > 2σ(*I*)
Mirror monochromator	*R*_int_ = 0.013
Detector resolution: 10.6501 pixels mm^-1^	θ_max_ = 70.1°, θ_min_ = 4.0°
ω scans	*h* = −6→6
Absorption correction: multi-scan (*CrysAlis PRO*; Agilent, 2011)	*k* = −12→12
*T*_min_ = 0.786, *T*_max_ = 1.000	*l* = −13→10
3789 measured reflections	

### Refinement

**Table:**

Refinement on *F*^2^	Primary atom site location: structure-invariant direct methods
Least-squares matrix: full	Secondary atom site location: difference Fourier map
*R*[*F*^2^ > 2σ(*F*^2^)] = 0.032	Hydrogen site location: inferred from neighbouring sites
*wR*(*F*^2^) = 0.088	H atoms treated by a mixture of independent and constrained refinement
*S* = 1.09	*w* = 1/[σ^2^(*F*_o_^2^) + (0.0466*P*)^2^ + 0.3904*P*] where *P* = (*F*_o_^2^ + 2*F*_c_^2^)/3
2341 reflections	(Δ/σ)_max_ < 0.001
193 parameters	Δρ_max_ = 0.30 e Å^−^^3^
0 restraints	Δρ_min_ = −0.39 e Å^−^^3^

### Special details

**Table:**

Geometry. All e.s.d.'s (except the e.s.d. in the dihedral angle between two l.s. planes) are estimated using the full covariance matrix. The cell e.s.d.'s are taken into account individually in the estimation of e.s.d.'s in distances, angles and torsion angles; correlations between e.s.d.'s in cell parameters are only used when they are defined by crystal symmetry. An approximate (isotropic) treatment of cell e.s.d.'s is used for estimating e.s.d.'s involving l.s. planes.
Refinement. Refinement of *F*^2^ against ALL reflections. The weighted *R*-factor *wR* and goodness of fit *S* are based on *F*^2^, conventional *R*-factors *R* are based on *F*, with *F* set to zero for negative *F*^2^. The threshold expression of *F*^2^ > σ(*F*^2^) is used only for calculating *R*-factors(gt) *etc*. and is not relevant to the choice of reflections for refinement. *R*-factors based on *F*^2^ are statistically about twice as large as those based on *F*, and *R*- factors based on ALL data will be even larger.

### Fractional atomic coordinates and isotropic or equivalent isotropic displacement parameters (Å^2^)

**Table:**

	*x*	*y*	*z*	*U*_iso_*/*U*_eq_	
N1	−0.2593 (2)	0.82964 (13)	0.67912 (12)	0.0146 (3)	
H1	−0.121 (4)	0.782 (2)	0.6983 (18)	0.018*	
C2	−0.3721 (3)	0.78622 (15)	0.58835 (14)	0.0147 (3)	
C3	−0.2635 (3)	0.66885 (16)	0.52306 (15)	0.0196 (3)	
H3	−0.1104	0.6194	0.5420	0.024*	
C4	−0.3861 (3)	0.62898 (17)	0.43183 (15)	0.0223 (4)	
H4	−0.3161	0.5514	0.3890	0.027*	
C5	−0.6203 (3)	0.70454 (17)	0.40110 (15)	0.0217 (4)	
H5	−0.6999	0.6760	0.3379	0.026*	
C6	−0.7286 (3)	0.81763 (17)	0.46292 (15)	0.0192 (3)	
H6	−0.8810	0.8660	0.4416	0.023*	
C7	−0.6090 (3)	0.86222 (15)	0.56042 (14)	0.0152 (3)	
N8	−0.7194 (2)	0.97138 (13)	0.62387 (12)	0.0170 (3)	
C9	−0.6022 (3)	1.00957 (15)	0.71603 (14)	0.0156 (3)	
C10	−0.7157 (3)	1.12367 (16)	0.78748 (15)	0.0195 (3)	
H10	−0.8713	1.1714	0.7708	0.023*	
C11	−0.5958 (3)	1.16250 (16)	0.88007 (15)	0.0211 (4)	
H11	−0.6713	1.2364	0.9269	0.025*	
C12	−0.3575 (3)	1.09182 (16)	0.90608 (15)	0.0209 (4)	
H12	−0.2782	1.1214	0.9688	0.025*	
C13	−0.2415 (3)	0.98128 (16)	0.84123 (15)	0.0186 (3)	
H13	−0.0857	0.9352	0.8597	0.022*	
C14	−0.3633 (3)	0.93855 (15)	0.74565 (14)	0.0142 (3)	
S1	0.28595 (6)	0.63836 (3)	0.83817 (3)	0.01403 (14)	
O1	0.1783 (2)	0.67614 (11)	0.72006 (10)	0.0209 (3)	
O2	0.3077 (2)	0.48835 (11)	0.85361 (11)	0.0193 (3)	
H2	0.155 (4)	0.469 (2)	0.8530 (18)	0.023*	
O3	0.1275 (2)	0.70674 (11)	0.93855 (11)	0.0214 (3)	
O4	0.5331 (2)	0.65395 (12)	0.84063 (12)	0.0247 (3)	
O1S	−0.0979 (2)	0.42771 (13)	0.84748 (11)	0.0207 (3)	
H1A	−0.221 (4)	0.498 (2)	0.8448 (19)	0.025*	
H1B	−0.120 (4)	0.387 (2)	0.912 (2)	0.025*	

### Atomic displacement parameters (Å^2^)

**Table:**

	*U*^11^	*U*^22^	*U*^33^	*U*^12^	*U*^13^	*U*^23^
N1	0.0135 (6)	0.0134 (6)	0.0161 (6)	−0.0012 (5)	−0.0020 (5)	0.0022 (5)
C2	0.0157 (7)	0.0149 (7)	0.0140 (7)	−0.0043 (6)	−0.0002 (6)	0.0024 (6)
C3	0.0195 (8)	0.0184 (8)	0.0189 (8)	−0.0002 (6)	−0.0016 (6)	−0.0003 (6)
C4	0.0276 (9)	0.0197 (8)	0.0190 (8)	−0.0043 (7)	−0.0006 (7)	−0.0036 (6)
C5	0.0248 (9)	0.0272 (9)	0.0158 (8)	−0.0108 (7)	−0.0044 (6)	−0.0006 (6)
C6	0.0170 (8)	0.0238 (8)	0.0179 (8)	−0.0062 (6)	−0.0041 (6)	0.0040 (6)
C7	0.0144 (7)	0.0163 (7)	0.0154 (7)	−0.0045 (6)	−0.0005 (6)	0.0033 (6)
N8	0.0152 (7)	0.0173 (7)	0.0183 (7)	−0.0030 (5)	−0.0017 (5)	0.0022 (5)
C9	0.0152 (7)	0.0147 (7)	0.0169 (7)	−0.0035 (6)	−0.0008 (6)	0.0028 (6)
C10	0.0182 (8)	0.0150 (8)	0.0229 (8)	0.0007 (6)	0.0006 (6)	0.0011 (6)
C11	0.0264 (9)	0.0137 (8)	0.0215 (8)	−0.0019 (6)	0.0011 (7)	−0.0012 (6)
C12	0.0276 (9)	0.0181 (8)	0.0183 (8)	−0.0074 (7)	−0.0039 (7)	−0.0013 (6)
C13	0.0184 (8)	0.0181 (8)	0.0196 (8)	−0.0040 (6)	−0.0048 (6)	0.0016 (6)
C14	0.0159 (7)	0.0115 (7)	0.0150 (7)	−0.0034 (6)	0.0007 (6)	0.0020 (6)
S1	0.0126 (2)	0.0130 (2)	0.0160 (2)	−0.00190 (14)	−0.00241 (14)	0.00158 (14)
O1	0.0199 (6)	0.0211 (6)	0.0178 (6)	0.0032 (5)	−0.0026 (5)	0.0026 (4)
O2	0.0169 (6)	0.0131 (6)	0.0272 (6)	−0.0019 (4)	−0.0025 (5)	0.0015 (4)
O3	0.0240 (6)	0.0181 (6)	0.0201 (6)	−0.0012 (5)	0.0001 (5)	−0.0022 (4)
O4	0.0172 (6)	0.0248 (6)	0.0338 (7)	−0.0080 (5)	−0.0048 (5)	0.0066 (5)
O1S	0.0175 (6)	0.0211 (6)	0.0223 (6)	−0.0022 (5)	−0.0007 (5)	0.0008 (5)

### Geometric parameters (Å, º)

**Table:**

N1—C2	1.342 (2)	C9—C14	1.431 (2)
N1—C14	1.347 (2)	C10—C11	1.360 (2)
N1—H1	0.86 (2)	C10—H10	0.9300
C2—C3	1.412 (2)	C11—C12	1.417 (2)
C2—C7	1.433 (2)	C11—H11	0.9300
C3—C4	1.363 (2)	C12—C13	1.366 (2)
C3—H3	0.9300	C12—H12	0.9300
C4—C5	1.428 (2)	C13—C14	1.410 (2)
C4—H4	0.9300	C13—H13	0.9300
C5—C6	1.359 (2)	S1—O4	1.4465 (12)
C5—H5	0.9300	S1—O3	1.4595 (12)
C6—C7	1.427 (2)	S1—O1	1.4685 (12)
C6—H6	0.9300	S1—O2	1.5452 (11)
C7—N8	1.340 (2)	O2—H2	0.93 (2)
N8—C9	1.345 (2)	O1S—H1A	0.89 (2)
C9—C10	1.427 (2)	O1S—H1B	0.84 (2)
			
C2—N1—C14	122.42 (14)	C10—C9—C14	118.10 (14)
C2—N1—H1	117.8 (13)	C11—C10—C9	119.91 (15)
C14—N1—H1	119.6 (13)	C11—C10—H10	120.0
N1—C2—C3	121.43 (14)	C9—C10—H10	120.0
N1—C2—C7	117.80 (14)	C10—C11—C12	120.87 (15)
C3—C2—C7	120.77 (14)	C10—C11—H11	119.6
C4—C3—C2	119.09 (15)	C12—C11—H11	119.6
C4—C3—H3	120.5	C13—C12—C11	121.57 (15)
C2—C3—H3	120.5	C13—C12—H12	119.2
C3—C4—C5	121.01 (15)	C11—C12—H12	119.2
C3—C4—H4	119.5	C12—C13—C14	118.48 (15)
C5—C4—H4	119.5	C12—C13—H13	120.8
C6—C5—C4	120.90 (15)	C14—C13—H13	120.8
C6—C5—H5	119.5	N1—C14—C13	121.24 (14)
C4—C5—H5	119.5	N1—C14—C9	117.71 (14)
C5—C6—C7	120.12 (15)	C13—C14—C9	121.05 (14)
C5—C6—H6	119.9	O4—S1—O3	113.27 (7)
C7—C6—H6	119.9	O4—S1—O1	112.32 (7)
N8—C7—C6	120.05 (14)	O3—S1—O1	110.75 (7)
N8—C7—C2	121.85 (14)	O4—S1—O2	104.85 (7)
C6—C7—C2	118.09 (14)	O3—S1—O2	107.93 (7)
C7—N8—C9	118.37 (14)	O1—S1—O2	107.28 (7)
N8—C9—C10	120.09 (14)	S1—O2—H2	111.1 (13)
N8—C9—C14	121.81 (14)	H1A—O1S—H1B	107 (2)
			
C14—N1—C2—C3	−177.42 (14)	C7—N8—C9—C10	−178.66 (14)
C14—N1—C2—C7	1.9 (2)	C7—N8—C9—C14	1.3 (2)
N1—C2—C3—C4	179.87 (15)	N8—C9—C10—C11	−179.55 (15)
C7—C2—C3—C4	0.6 (2)	C14—C9—C10—C11	0.5 (2)
C2—C3—C4—C5	0.5 (3)	C9—C10—C11—C12	0.7 (2)
C3—C4—C5—C6	−0.7 (3)	C10—C11—C12—C13	−1.3 (3)
C4—C5—C6—C7	−0.3 (3)	C11—C12—C13—C14	0.6 (2)
C5—C6—C7—N8	−177.83 (15)	C2—N1—C14—C13	179.42 (14)
C5—C6—C7—C2	1.4 (2)	C2—N1—C14—C9	−0.6 (2)
N1—C2—C7—N8	−1.6 (2)	C12—C13—C14—N1	−179.43 (15)
C3—C2—C7—N8	177.64 (15)	C12—C13—C14—C9	0.6 (2)
N1—C2—C7—C6	179.15 (13)	N8—C9—C14—N1	−1.1 (2)
C3—C2—C7—C6	−1.6 (2)	C10—C9—C14—N1	178.86 (14)
C6—C7—N8—C9	179.27 (14)	N8—C9—C14—C13	178.92 (14)
C2—C7—N8—C9	0.1 (2)	C10—C9—C14—C13	−1.1 (2)

### Hydrogen-bond geometry (Å, º)

**Table:**

*D*—H···*A*	*D*—H	H···*A*	*D*···*A*	*D*—H···*A*
N1—H1···O1	0.86 (2)	1.81 (2)	2.6685 (18)	173.3 (19)
O2—H2···O1*S*	0.93 (2)	1.59 (2)	2.5223 (16)	177 (2)
O1*S*—H1*A*···O4^i^	0.89 (2)	1.87 (2)	2.7577 (18)	176 (2)
O1*S*—H1*B*···O3^ii^	0.84 (2)	1.90 (2)	2.7405 (18)	173 (2)
C6—H6···N8^iii^	0.93	2.61	3.538 (2)	172

Symmetry codes: (i) *x*−1, *y*, *z*; (ii) −*x*, −*y*+1, −*z*+2; (iii) −*x*−2, −*y*+2, −*z*+1.
